# Reliability and validity of pediatric triage tools evaluated in Low resource settings: a systematic review

**DOI:** 10.1186/s12887-017-0796-x

**Published:** 2017-01-26

**Authors:** Bhakti Hansoti, Alexander Jenson, Devin Keefe, Sarah Stewart De Ramirez, Trisha Anest, Michelle Twomey, Katie Lobner, Gabor Kelen, Lee Wallis

**Affiliations:** 1 Department of Emergency Medicine, Johns Hopkins School of Medicine, 1830 Monument St Suite 6-100, Baltimore, MD 21287 USA; 2 University of Cape Town Division of Emergency Medicine, Private Bag X24, Bellville, 7535 South Africa; 3 Welch Medical Library, Johns Hopkins School of Medicine, Baltimore, USA

## Abstract

**Background:**

Despite the high burden of pediatric mortality from preventable conditions in low and middle income countries and the existence of multiple tools to prioritize critically ill children in low-resource settings, no analysis exists of the reliability and validity of these tools in identifying critically ill children in these scenarios.

**Methods:**

The authors performed a systematic search of the peer-reviewed literature published, for studies pertaining to for triage and IMCI in low and middle-income countries in English language, from January 01, 2000 to October 22, 2013. An updated literature search was performed on on July 1, 2015. The databases searched included the Cochrane Library, EMBASE, Medline, PubMed and Web of Science. Only studies that presented data on the reliability and validity evaluations of triage tool were included in this review. Two independent reviewers utilized a data abstraction tool to collect data on demographics, triage tool components and the reliability and validity data and summary findings for each triage tool assessed.

**Results:**

Of the 4,717 studies searched, seven studies evaluating triage tools and 10 studies evaluating IMCI were included. There were wide varieties in method for assessing reliability and validity, with different settings, outcome metrics and statistical methods.

**Conclusions:**

Studies evaluating triage tools for pediatric patients in low and middle income countries are scarce. Furthermore the methodology utilized in the conduct of these studies varies greatly and does not allow for the comparison of tools across study sites.

**Electronic supplementary material:**

The online version of this article (doi:10.1186/s12887-017-0796-x) contains supplementary material, which is available to authorized users.

## Background

The global burden of pediatric mortality in low resource settings remains high; 6.3 million children under five years old die worldwide each year. Although under-five mortality has declined from 90 to 43 deaths per 1,000 live births since 1990, improvements have fallen short of Millennium Development Goal (MDG) 4 which called for a two-thirds reduction in mortality worldwide by 2015 [1]. A majority of childhood deaths are attributable to easily treatable, time sensitive illness [2]. It is estimated that as much as 60% of mortality in this population may be reduced by improving access to care [3]. Providing timely access to specialized emergency care has been shown in numerous settings to confer a mortality benefit [4].

Triage is the prioritization of patients, usually to identify the sickest for earliest intervention; it typically consists of a complex decision-making process including clinical discriminators, physiological parameters, or both [5]. Triage has the ability to substantially decrease pediatric mortality and morbidity by providing timely care for critically ill patients [6]. Several validated scales exist; however, much of the triage data is derived from high-income countries [7].

In recent years, there has been a push to develop triage scales specifically tailored to low resource environments in Low and Middle Income Countries (LMICs) [8]; examples include tools such as the Pediatric South African Triage Scale (PSATS) [9] and the WHO Emergency Triage and Treatment Tool (ETAT) [10] among others. In the clinic setting healthcare workers utilize the WHO developed and implemented Integrated Management of Childhood Illnesses (IMCI) to identify pediatric patients with time sensitive illness requiring urgent treatment and/or referral [2, 3].

Although not a traditional triage tool, the IMCI is a well studied and widely implemented in both out patient clinic and hospital settings. Therefore, the authors’ felt that studies evaluating the IMCI in LMICs warranted specific consideration. It is imperative that healthcare providers and policy makers understand the evidence and generalizability of the evaluation studies of these tools, among others, prior to implementation. This systematic review aims to investigate the scientific evidence underlying the use of acute care triage scales and IMCI for pediatric patients in LMICs.

## Methods

### Search strategy

The authors performed a systematic search of the peer-reviewed literature published, in English language, from January 01, 2000 to October 22, 2013, with an updated literature search on July 1, 2015. The databases searched included the Cochrane Library, EMBASE, Medline, PubMed and Web of Science.

Two separate searches were conducted. Both searches included search filters for LMICs (Additional file 1: Appendix A). The first search included the Medical Subject Heading (MESH) term “triage”, and a separate search was conducted for the Integrated Management of Childhood Illness (IMCI). Income status of the country was defined by World Bank criteria [11]. All applicable controlled vocabularies and keyword terms were searched in all databases. The search was run without any restrictions and two authors screened each result. All studies pertaining to the evaluation of triage tools for pediatric patients (<18 years of age), in an acute care setting (i.e., where undifferentiated patients present for care), were included in the review. We included studies conducted in both hospitals and clinic settings.

Studies were excluded if the described tool was not designed to affect patient treatment or destination (i.e. a trauma score), if the tool was disease specific, or if the article was not available in English. Various study designs were included such as randomized control trials, observational studies and descriptive studies, however case reports or case series (defined as *n* < 5) were, excluded (Additional file 2: Appendix B).

Each study included in the review underwent data abstraction using a data abstraction tool (Additional file 3: Appendix C) by two independent reviewers. We collected data on four elements, (1) the demographics of the study locale, (2) the triage tool components, (3) the reliability data and (4) the validity data and summary findings for each triage tool assessed. Evaluations of the studies, including the risk of bias or an evaluation of quality of the individual study methodology, was not a component of the data abstraction tool for this review.

### Outcome variables

Reliability was defined as the assessment of triage tools against other evaluations, either by another health care professional (inter-rater), or a triage tool expert designer/study author (expert opinion). Validity was defined as evaluation of outcomes for triaged patients (admission, ICU admission, referral, death etc.) by triage category. Multiple studies included assessments of “over” and “under” triage, but given the heterogeneity of these definitions [12, 13] and different methods of computing the result across studies [13, 14], these analyses were not included in the review.

## Results

**Fig. 1:**
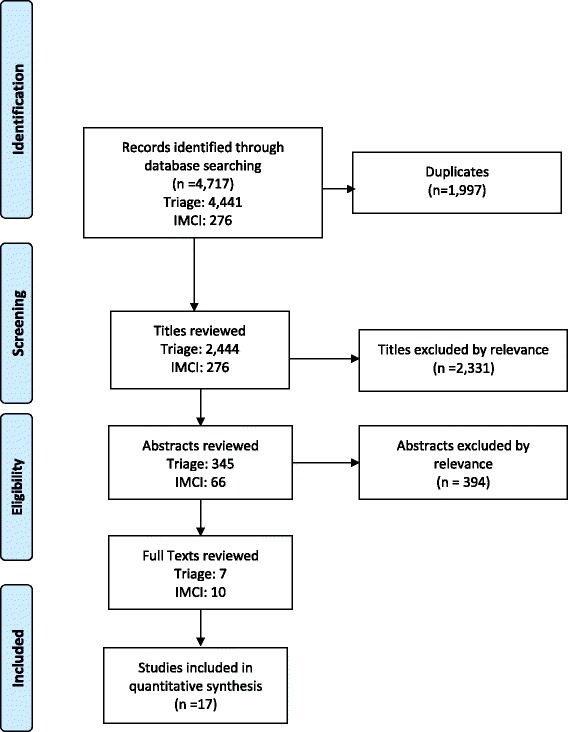
PRISMA flow diagram of search methodology

The initial search strategy returned 4,717 results, with 2,742 unique articles (Fig. 1). Each title was then assessed for inclusion based on the specific criteria above and was analyzed by two independent reviewers; a third senior author evaluated articles with discordant results.

After the initial title review, 411 abstracts were identified, including triage of both adult and pediatric patients. A total of seven studies evaluating triage tools in pediatric patients and 10 studies evaluating IMCI are included in the review.

### Study locale

Seven studies assessed a total of five triage tools in pediatric acute care settings in LMICs. Only one of the tools (ETAT) was evaluated in a low income country (Malawi) [15]. All of the remaining tools were evaluated in middle-income countries, with four tools (PEWS, PSATS, ETAT, ESI) [10, 16–18] exclusively evaluated in upper-middle income countries. Five studies were conducted in tertiary care centers, and one study was conducted in a district hospital setting [10]. Only one study was multi-center, with Twomey et al [18] evaluating PSATS in 5 hospitals and 1 community health center.

IMCI evaluations were exclusively conducted in lower middle-income countries (India, Bolivia, and Vietnam) [19–28]. One of the ten studies was conducted in exclusively outpatient settings [28], five were conducted in hospital EDs [19–22, 26], and four were conducted in both settings as a multi-center evaluation.

#### Tool components

The tools included for pediatric triage had varying components, and as a result varying levels of complexity (Table 1). The ETAT guidelines involve triage of patients according to emergency and priority signs using an ABCD concept (Airway, Breathing, Circulation/Coma/Convulsion, Dehydration), and rely on clinical discriminators rather than physiologic parameters to stratify sick children [10]. Conversely, the PEWS (Pediatric Early Warning Score) and TOPRS (Temperature, O2 saturation, Pulse, Respiratory rate, Sensorium/Seizures) relies solely on physiologic parameters (vital signs) to predict hospitalization [16, 29]. Some tools combined both physiologic parameters and clinical discriminators such as the Pediatric South African Triage Scale (pSATS) which incorporates the ETAT ABCD emergency signs as well as Triage Early Warning Score (TEWS) physiologic parameters to stratify patients. Lastly, the Emergency Severity Index (ESI) adds further complexity by asking the provider the number of resources that will be required.

**Table 1:** Overview of study local, study type and triage tool components included in the systematic review

Tool	Studies	Evaluations	Country Setting		Study Locale			Study Type		Tool Components			
			Middle Income	Low Income	Tertiary Hospital	District Hospital	Outpatient Clinic	Reliability	Validity	Vital Signs	Clinical Discriminators	Presenting Complaint	Resources Required
pSATS^a^	2	3	x		x		x		x	x	x	x	
ETAT^b^	2	2	x	x	x	x	x	x	x		x		
ESI	1	2	x		x			x	x	x	x	x	x
PEWS	1	4	x		x			x	x		x		
TOPRS	1	1	x		x				x	x			
IMCI	10	12	x		x	x	x	x	x	x	x	x	

^a^Includes both pSATS and PATS, a modification of SATS with minor changes

^b^Includes both ETAT and abbreviated ETAT

IMCI utilizes a syndrome-based approach to target the care of children, and thus uses physiologic and clinical discriminators in evaluating triage category. This is because, although the IMCI strategy does have a prioritization component (so that critically ill children may be transferred to a higher level of care), its stated purpose is to promote curative care in the outpatient setting, and includes algorithms for healthcare workers to direct care for common complaints in a pediatric outpatient settings.

### Pediatric triage tools

#### Reliability

Three studies measured the reliability of three different triage tools (Table 2) [7, 16, 17]. Roberston et al. evaluated ETAT in Malawi and found that nurses have an agreement of 93.8%, assigning the correct triage level, when compared against physician “assessors” observing triage [7]. The ESI evaluation by Jafari-Rouhi et al. utilized blinded re-rating by pediatric emergency medicine physicians to assess nursing performance with an overall kappa of 0.82 [17]. Both studies included real patients with real-time triage evaluations in a clinical setting, and all studies were appropriately blinded to the other provider’s assessment. The highest percentage agreement was achieved in the level 1/priority 1 patient group.

**Table 2:** Included reliability studies of pediatric triage tools

Triage Tool	Author[ref] Year Country (Income level)	Comparison Groups	Volume Patient and Setting Characteristics (Age restrictions)	Results (kappa, percent agreement) by triage level
ETAT	Robertson etal. [7] 2001 Malawi (Low Income)	1. Clinic nurse 2. ED physician	*n* = 2281 Under 5 outpatient clinic (85% <5y/o)	Overall: 94.8% P1: 95.7% P2: 88.0% P3: 96.1%
ESI	Jafari-Rouhi etal. [17] 2013 Iran (Upper Middle Income)	1. Triage nurse to Ped 2. ED physician	*n* = 1104 Emergency department at national teaching hospital (100% <18 y/o)	Kappa 0.82 Overall 87.3% Level 1: 100% Level 2: 93.1% Level 3: 83.4% Level 4: 86.1% Level 5: 84.1%
PEWS	Chaiyakulsil etal. [16] 2015 Thailand (Upper Middle income)	1. Triage nurse 2. Triage nurse	*n* = 1136 ED at large tertiary care hospital	Kappa: 0.75

#### Validity

All seven triage studies included some measurement of validity [4, 7, 10, 16–18]. The studies included, tools evaluated, and outcome measures utilized are shown in Table 3.

**Table 3:** Included validation studies of pediatric triage tools

Outcome	Scale	Author [ref] Year Country (Income)	Site Volume Patient and Setting Characteristics (Age restrictions)	Results (per triage level) [*p* value]	Remarks
Mortality	ESI	Jafari-Rouhi [17] 2013 Iran (Upper Middle Income)	*n* = 1104 ED at national teaching hospital. (100% <18 y/o)	Overall: 0.9% 1: 100% 2: 1.2% 3:0% 4:0% 5: 0%	Outcome was ICU admission or death. ESI performed at patient presentation, not admission.
	TOPRS	Bains 2012 [15] India (Lower-middle Income)	*n* = 777 Teaching hospital with general ED.	Overall: 16.3% TORPS 6: 100% TORPS 5: 80.0% TORPS 4: 66.7% TORPS 3: 60.0% TORPS 2: 38.2% TORPS 1: 12.5% TOPRS 0: 4.4%	All patients were admitted to ED. ROC curve maximal discrimination at 2.5 (sensitivity 79.6%, specificity 74.3%)
	PATS	Mullan 2014 [ ] Botswana (Upper-middle Income)	*n* = 4466 ED at terteriary referral hospital (100% < 13 y/o)	Overall: 0.16% Red: 1.4% Orange: 0.05% Yellow: 0% Green: 0%	Outcome was ICU admission or death. PATS performed at ED presentation. Large study of both adult and pediatric patients with separate analyses.
Admission	ESI	Jafari-Rouhi 2013 [17] Iran (Upper-middle Income)	*n* = 1104 ED at national teaching hospital. (100% <18 y/o)	Overall: 9.4% 1: 0% 2: 29.8% 3: 1.8% 4: 2.0% 5: 0%	Outcome was ED admission or ward admission (does not include ICU admission). Spearman correlation coeficient 0.407.
	Adapted ETAT	Buys 2013 [10] South Africa (Upper-middle Income)	*n* = 407 District hospital with general ED. (100% < 16y/o)	Overall: 24.8% P1: 91.7% P2: 36.9% P3: 10.1%	Second of 2 cohorts (2009), first (2007) immediately following training.
	pSATS	Twomey 2013 [18] South Africa (Upper-middle Income)	*n* = 2014 6 ED centers with varying size/populations. (100% <13 y/o)	Overall: 21.5% 1: 72.8% 2: 29.0% 3: 27.9% 4: 4.7%	Sensitivity 91.0%, Specificity 54.5%. Compared to simply TEWS or clinical discriminator, and improved discrimination.
	ETAT	Robertson 2001 [7] Malawi (Low Income)	*n* = 2281 Under 5 outpatient clinic with referral for admission (85% <5 y/o)	Overall: 14.9% P1: 90.0% P2: 32.0% P3: 4.5%	No follow-up data after admission. Only patients under 5.
	PEWS	Chaiyakulsil 2015 [16] Thailand (Upper-Middle income)	*n* = 1136 ED at large tertiary hospital (100% < 15y/o)	Overall AUC: 0.73 ICU: 0.98 Ward 0.71 PEWS > 1 for admission Sensitivity 77% Specificity 59%	Measured in area under ROC curve, for sensitivity and specificity for admission by PEWS category.
	PATS	Mullan 2014 [ ] Botswana (Upper-middle Income)	*N* = 4466 ED at terteriary referral hospital (100% < 13 y/o)	Overall: 54.5% Red: 86.7% Orange: 66.1% Yellow: 37.6% Green: 20.6%	PATS performed at ED presentation. Large study of both adult and pediatric patients with separate analyses.

Two studies analyzed the likelihood of admission by ETAT triage level assigned. Ninety percent of priority 1 (P1 or critically ill) patients were admitted versus 32% of P2 and 4.5% of P3 in one study [7]. Another group published similar percentages with a significant increase in the relative risk of admission in children triaged to level 1 or 2 compared to 3 (RR 2.6; 95% CI 2.2-3.1 in one sample and RR 3.2; 95% CI 2.5-4.1 in another) [10]. Using the pSATS tool, Twomey et al. reported an increase in hospital admission with increasing level of urgency from 4.7% in the lowest triage level to 72.8% of those triaged to level 1 [18]. The area under the ROC curve for predicting overall admission using PEWS was 0.73. PEWS was 100% sensitive and 90.5% specific for predicting ICU admission [16]. In a large study conducted by Mullan et al. analyzed both adult and pediatric (<13 years old) patients using PATS, a modified form of SATS. They demonstrated an increase in hospital admission (20.6–86.7%) and mortality (0–1.4%) when children were assigned a higher acuity score [30, 31].

Using the ESI v.4 in Iran, 100% of patients assigned level 1 by pediatric EM physicians were either admitted to the ICU or died while 0% were admitted to the ward or discharged [17]. Of level 2 patients, 1.2% were admitted to the ICU or died, 29.8% were admitted to the ward, and 69% were discharged. Zero level 3 through 5 patients went to the ICU or died [17]. The TOPRS score was found to have a predictive ability of 81.7% for admission on receiver operating curve analysis with a progressive increase in mortality by increasing score.

### IMCI/IMNCI

#### Reliability

Six of the included studies published Kappa or percent agreement data to quantify the concordance of nurse or health worker IMCI determination with physician diagnosis (Table 4) [19–23, 28]. There was a large variation in the overall Kappa values, from 0.16 to 0.59 [19, 26]. Bhattacharya et al., 2011, reported a syndrome specific kappa and found the highest agreement in the diagnosis of jaundice (0.73) and the lowest agreement in the diagnosis of dehydration (0.19) [21].

**Table 4:** Included Evaluations of Reliability of IMCI/IMNCI

Type of Reliability Evaluation	Author[ref] Year Country (Income level)	Comparison Groups	IMCI Variable Being Assessed	Volume Patient and Setting Characteristics (Age restrictions)	Results (kappa (k), percent agreement (Pa), Sensitivity (Sn), Specificity (Sp)) by triage level
Inter-Rater^a^	Shewade [24] 2013 India (Lower-Middle Income)	1. Health Care Worker 2. Study Investigator	IMCI category	*N* = 128 Community health center (0mos-5 yrs)	Overall k = 0.43 <2mos k = 0.31 >2mos k = 0.44
	Battarachaya [21] 2012 India (Lower-Middle Income)	1. Health Care Worker 2. Physician (IMCI Red/Yellow)	IMCI Red/Yellow categorization	*n* = 131 Inpatient pediatric hospital (2mos-5 yrs)	Pa = 36% Simple k = 0.16 Weighted k = 0.29
	Battarchaya [20] 2013 India (Lower-Middle Income)	1. Health Care Worker 2. Physician (Diagnosis)	Diagnostic category	*n* = 117 Inpatient pediatric hospital (0-2mos)	Serious Bacterial Infection k = 0.38 Local Bacterial Infection k = 0.20 Jaundice k = 0.73 Dehydration k = 0.19 Unable to Feed k = 0.29
	Battarchaya [19] 2011 India (Lower-Middle Income)	1. Health Care Worker 2. Physician (IMCI Red/Yellow)	IMCI Red/Yellow categorization	*n* = 117 Inpatient pediatric hospital (0-2mos)	Pa = 56% Simple k = 0.32 Weighted k = 0.41
	Gupta [23] 2000 India (Lower-Middle Income)	1. Health Care Worker 2. Physician	IMCI Categorization	*n* = 129 ED and Outpatient clinics (0-2mos)	Pa = 60%
Gold Standard Comparison^b^	Mazzi [26] 2010 Bolivia (Lower-Middle Income)	1. Triage Nurse 2. ED Physician^a^	IMNCI Red within Diagnostic categories	*n* = 1082 2 pediatric hospital ED (0mos-2mos)	Sn <35% for each sign Sp >85% for all signs
	Battarachaya [20] 2013 India (Lower-Middle Income)	1. ED Nurse 2. Inpatient Physician^a^	IMNCI Red within Diagnostic categories	*n* = 117 Inpatient pediatric hospital (0-2mos)	Serious bacterial infection: 89% Sn, 72% Sp Local Bacterial Infection: 14% Sn, 99% Sp Jaundice: 67% Sn, 99% Sp Dehydration: 25% Sn, 95% Sp Poor feeding: 44% Sn, 87% Sp
	Mittal [27] 2013 India (Lower-Middle Income)	1. Health Care Worker 2. ED Physician^a^	IMCI Red categorization	n = 1043 Outpatient and pediatric ED in tertiary care center (0mos-5 yr)	38.7% diagnostic mismatch in 0-7d age group 24.3% diagnostic mismatch in 7d-2mos age group 19.9% mismatch in 2mos-5 yr
	Cao [22] 2004 Vietnam (Upper-Middle Income)	1. Health Care Worker 2. Physician^a^	IMCI Red categorization	*n* = 859 Inpatient pediatric hospital (2mos-5 yrs)	Severe Illness (IMCI Red) Sn 94.7% Sp 96.1%

^a^Inter-Rater reliability was measured as 2 individuals agreement without weight of importance. It is expressed in percent agreement or kappa statistics

^b^Gold Standard Comparison expresses the ability of triage personnel (workers, nurses) to physicians. It is expressed in sensitivity and specificity

#### Validity

Six studies evaluated the validity of IMCI (Table 5) [19, 20, 24–27]. One study showed an increase in likelihood of admission according to urgency of triage level assigned [19]. Mazzi et al., reported that the sensitivity of individual clinical signs for predicting serious illness requiring hospital management was less than 35% for all signs except fever (65%) [26].

**Table 5:** Included evaluations of validity by real patient outcomes of IMCI/IMNCI

Outcome	Author [ref] Year Country (Income)	Site Volume Patient and Setting Characteristics (Age restrictions)	Results (per triage level) [*p* value]
Admission	Battarachaya [19] 2012 India (Lower-Middle Income)	*n* = 131 Inpatient pediatric hospital ED (2mos-5 yrs)	Overall: 25% Red: 82% Yellow: 11% Green 5%
Referral to ED	Kaur [24] 2011 India (Lower-Middle Income)	*n* = 419 Outpatient pediatric clinic and ED in tertiary care center (0-2mos)	95% Sn 87% Sp
	Kundra [25] 2008 India (Lower-Middle Income)	*n* = 309 Outpatient pediatric clinic and ED in tertiary care center (2mos-5 yrs)	98% Sn

## Discussion

Overall, the quantity and quality of evidence to support the effectiveness of any single triage tool for pediatric patients in low resource settings is poor. This is driven by the limited number of studies available; the heterogeneous nature of these studies preventing formal meta-analysis; and the high proportion of studies conducted in urban centers in middle-income countries preventing extrapolation to low resource environments.

Our study identified a research gap in the quality and quantity of studies conducted in urban middle-income countries compared to rural environments in low-income countries, true low resource settings. Only one of the 16 studies were carried out in a low-income country [15] (Table 1). Although it is much more feasible to evaluate triage tools in high resourced district and tertiary care hospitals in middle-income countries (many of which resemble hospitals in high-income countries), these studies are difficult to extrapolate to rural low resource settings, where the need for these tools is greatest. In addition one may hypothesize that the triage tool may alter in their function in low resource settings given differences in available treatments, training of providers, underlying healthcare system infrastructure and prevalent disease pathologies (i.e., high prevalence of HIV infection and malnutrition for example).

The tools included in this study varied in their construct. ETAT was an example of the use of clinical signs alone, without vital signs or any input of the presenting complaint. Tools based on clinical signs alone have appeal as they can be employed quickly in settings where measuring vital signs may be too time-consuming or impractical [10]. Conversely PEWS and TOPRS allow for a true objective provider assessment, which may be less likely to introduce bias and may be performed by providers with more basic training [16, 29]. Then, tools such as the pSATS and PATS incorporate both clinical and physiologic data. The Pediatric South African Triage Scale (pSATS), incorporates the ETAT ABCD emergency signs as well as Triage Early Warning Score (TEWS) physiologic parameters to stratify patients. Twomey et al. studied the sensitivity of the pSATS tool, suggesting it is a more robust screening modality than either clinical discriminators or TEWS alone. The sensitivity (Sn) and negative predictive value (NPV) of pSATS was higher (91 and 93%, respectively) when compared to clinical discriminators alone (Sn 57 and NPV 86%) or TEWS alone (Sn 75.6 and NPV 89%). Appropriately, children triaged to lowest category were correctly identified as non-urgent. Advocates of these mixed triage scores argue that the addition of vital signs significantly increases the sensitivity for identifying sick children and outweighs the additional time required [18].

Our original intent was to make comparisons between triage tools, and to meta-analyze the reliability and validity of tools in different settings. This was impossible due to the limited number of studies, and variability in study design. Reliability assessments varied in statistical analyses (from kappa statistics to percent agreement [15] to sensitivity/specificity of a binary outcome [22, 26]), and methodology (comparison groups varied between studies, IMCI variable varied in reliability assessments) (Table 1, Table 3). This made formal meta-analysis impossible, and prevented a true global assessment of the reliability of any one triage tool.

A number of studies reported nursing triage to physician triage [17]. However the use of physicians as a gold standard in triage sensitivity does also give cause of concern. In most developed countries, a nursing professional is responsible for triage operations. Given the lack of healthcare provider resources, physicians in triage would be very unusual in LMICs. Therefore, it is unclear if the use of a physician as the gold standard for reliability measurements is appropriate in these studies. All reliability studies did however utilize real patients in their evaluations opposed to written scenarios, and may be the reason for poor reliability data that is reported. In adult patients the many reliability studies use pre fabricated written cases to assess reliability and thus report higher agreement [32, 33].

Validity studies also varied widely, where different methodologies prevented true meta analysis and study limitations hindered the quality of evidence. All validity studies had large sample sizes, ranging from 407 [10] to 4466 [31] participants. However, the methodology varied widely, with some triage applying to patients who were “admitted” to the ED (ie were expected to stay for a period of time) [29], while others were done at initial presentation. A major source of variability was the overall rates of mortality and admission at different locales. The studies measuring admission outcomes differed significantly in their overall admission rates from 9.4% [34] to 55.4% [31]. Overall mortality at the study sites varied from 0.16% [31] to 16.3% [29].

Most studies rely on admission or mortality as a proxy for severity of illness. In low resource health care environments, there are numerous confounders that can impact outcomes including the training of providers, availability of medications and surgical interventions, availability of specialty/critical care, and the ability of patients to pay for treatment. In addition, the lack of follow-up data in any of these studies significantly hinders its effectiveness. For those patients not admitted, there is absolutely no data on mortality or re-presentation in any of the studies featured. Given resource and infrastructure constraints (census, patient records etc.), this is an understandable, but significant, limitation of the research in this field. Furthermore, this oversight is not merely restricted to studies in LMICs but also a limitation of several of the studies in the Farrokhnia review that focuses on high resource settings [5]. A systematic effort is required to overcome this limitation. Funders and investigators need to prioritize prospective evaluations with an emphasis on follow up of all patients that are triaged during the study period opposed to retrospective evaluations that only include admitted patients.

A single study by Molyneaux et al., does warrant special mention [6]. They demonstrated a near 50% reduction in under five-year-old inpatient mortality at a district hospital in Malawi after making improvements in triage, which included formal ETAT training. Although, in its true essence, this study does not evaluate the validity of a triage tool, the authors demonstrated that implementation of a triage system in their clinical environment significantly reduced overall child mortality. This study’s validity cannot be appraised, as the authors do not provide specific information on the outcome of patients assigned to various categories. In addition, the pre/post study design is prone to multiple confounders given the many simultaneous changes to the triage system (new hires, better clinician oversight, new physical plant, etc.). These confounders make quantifying the effects of ETAT alone impossible [6].

In evaluating the evidence supporting IMCI, it is evident that, although numerous, there is insufficient evidence to validate the tool in varied low-resource environments, due to similar problems with heterogeneous study methods and numerous study limitations. Comparing IMCI to other pediatric triage tools, IMCI also addresses aspects of nutrition, immunization, and other important elements of disease prevention and health promotion. Accordingly, many of the IMCI studies considered in this review evaluate patients in an outpatient or clinic setting. Interestingly, the volume of studies evaluating IMCI far outnumber the literature on any of the triage tools, likely due to the 1994 WHO mandate to complete large multi-country evaluations on the training and implementation of IMCI worldwide [35].

Despite the relative plethora of studies validating IMCI, there are significant limitations in study design and locale. First, the IMCI studies are smaller in size, likely representing the relative patient census at the smaller centers in which the studies were conducted. In addition, these studies suffer from a lack of standard method for assessing IMCI reliability, and individual studies present varied methods of comparison, comparison groups, and IMCI variables to be compared (Table 3). This prevents a formal analysis of the overall IMCI reliability between raters.

Additionally, the kappa values for reliability are significantly lower than those reported in the triage tool studies (Table 3). One possible explanation is that IMCI is typically utilized by health care providers with less formal training, while the responsibility of triage in larger centers is often placed on professional nurses with formal schooling. In addition, numerous studies attempt to make validity conclusions based on IMCI performance compared to physician diagnosis as gold standard. In India, Kaur and others demonstrated that the IMNCI adaptation is a sensitive tool (95%) for identifying neonates for referral [25, 36] It is concerning that so many studies opted to use non blinded physician opinion as the gold standard. Diagnostic agreement and decision to admit or refer are generally poor metrics for validation, given that they are inherently biased by the initial triage decision. Of the studies considered in this review, none followed patients to collect outcome data such as treatments required, length of stay, or mortality [20]. Taken cumulatively, all of these limitations prevent a formal analysis of the reliability and validity of IMCI, and thus limit the ability to recommend it for practitioners in low-resource settings.

### Limitations

This review only includes studies that were published in the peer-reviewed literature available on databases searched. Second, other tools that may be used to prioritize the care for children in low-resource settings may not be referred to as “triage” tools. Recognizing this, authors performed a separate search for IMCI, but other similar approaches may exist that were not included. Studies not available in English language were not considered in this review. Relevant studies published without translation may have been excluded.

## Conclusions

Overall, there is little in the literature studying the performance of triage tools in pediatric patients in low resource settings. The generalizability of these studies is also difficult given the preponderance of studies conducted in urban centers in middle-income countries opposed to true low resource settings. A large number of studies depend on the local physician assessment as a gold standard, which is highly variable and difficult to reproduce across studies. Thus it is difficult to support the use of a single tool based on this systematic review. Despite the methodological concerns evaluating IMCI studies, the ubiquitous use of IMCI as well as the availability of training and implementation though the WHO does support its continued use in outpatient clinic settings where it is currently implemented.

Overall studies evaluating triage tools in this vulnerable population are scarce and generally do not include follow up of lower acuity patients and critically important outcomes data. There is a need to develop and define robust validation methodology that can be prospectively utilized to evaluate triage tools in low resource settings.

## Additional files

Additional file 1: Appendix A Search Strategy. (DOCX 24 kb)
Additional file 2: Appendix B. Systematic review inclusion and exclusion criteria. (DOCX 71 kb)
Additional file 3: Appendix C. Triage Tools Data Abstraction. (PDF 126 kb)

## Abbreviations

ED: Emergency department
ESI: Emergency severity index
ETAT: Emergency triage and treatment tool
ICU: Intensive care unit
IMCI: Integrated management of childhood Illness
LMIC: Low and middle income countries
MDG: Millennium development goal
NPV: Negative predictive value
PEWS: Pediatric early warning score
PSATS: Pediatric south african triage scale
Sn: Sensitivity
TEWS: Triage early warning score
TOPRS: Temperature, O2 saturation, pulse, respiratory rate, sensorium/seizures
WHO: World health organization
