# Supplementary material for: Reliability and validity of pediatric triage tools evaluated in Low resource settings: a systematic review
Source: BMC Pediatr. 2017 Jan 26;17:37. doi: 10.1186/s12887-017-0796-x (PMC5267450; doi:10.1186/s12887-017-0796-x)
Supplement: Additional file 1: Appendix A — Search Strategy. (DOCX 24 kb) [file 12887_2017_796_MOESM2_ESM.docx]

**Appendix A: Systematic review inclusion and exclusion criteria**

**Inclusion criteria:**

- Studies evaluating reliability of triage scales
- Studies evaluating performance of scales on real patients triaged in the ED (validity)
- Studies evaluating mortality and outcome by triage level (validity)
- Implementation analysis of triage scales
- Both fictitious and real patient scenarios
- Evaluation of sensitivity/specificity/over/under triage
- Studies evaluating specific physiologic parameters rather than composite scores

**Exclusion criteria:**

- Adult patients (>18 y/o)
- High income countries
- Case studies (N<5)
- Non English/No Abstract
- Published prior to January 1, 2000
- Tools that do not designed to directly affect patient treatment or destination (ie trauma scores)
- Tools used for specific complaints or diseases (ie pneumonia, respiratory distress).
