# Supplementary material for: Reliability and validity of pediatric triage tools evaluated in Low resource settings: a systematic review
Source: BMC Pediatr. 2017 Jan 26;17:37. doi: 10.1186/s12887-017-0796-x (PMC5267450; doi:10.1186/s12887-017-0796-x)
Supplement: Additional file 2: Appendix B. — Systematic review inclusion and exclusion criteria. (DOCX 71 kb) [file 12887_2017_796_MOESM1_ESM.docx]

**Appendix B Search Strategy:**

Pubmed search strategy (coverage 1946-April 23, 2015):

("Triage"[mh] OR "triage" OR "triaged")

AND

("developing country"[tiab] OR "developing countries"[tiab] OR "developing nation"[tiab] OR "developing nations"[tiab] OR "developing population"[tiab] OR "developing populations"[tiab] OR "developing world"[tiab] OR "less developed country"[tiab] OR "less developed ountries"[tiab] OR "less developed nation"[tiab] OR "less developed nations"[tiab] OR "less developed population"[tiab] OR "less developed populations"[tiab] OR "less developed world"[tiab] OR "lesser developed country"[tiab] OR "lesser developed countries"[tiab] OR "lesser developed nation"[tiab] OR "lesser developed nations"[tiab] OR "lesser developed population"[tiab] OR "lesser developed populations"[tiab] OR "lesser developed world"[tiab] OR "under developed country"[tiab] OR "under developed countries"[tiab] OR "under developed

nation"[tiab] OR "under developed nations"[tiab] OR "under developed population"[tiab] OR "under developed populations"[tiab] OR "under developed world"[tiab] OR "underdeveloped country"[tiab] OR "underdeveloped countries"[tiab] OR "underdeveloped nation"[tiab] OR "underdeveloped nations"[tiab] OR "underdeveloped population"[tiab] OR "underdeveloped

populations"[tiab] OR "underdeveloped world"[tiab] OR "middle income country"[tiab] OR "middle income countries"[tiab] OR "middle income nation"[tiab] OR "middle income ations"[tiab] OR "middle income population"[tiab] OR "middle income populations"[tiab] OR "low income

country"[tiab] OR "low income countries"[tiab] OR "low income nation"[tiab] OR "low income nations"[tiab] OR "low income population"[tiab] OR "low income populations"[tiab] OR "lower income country"[tiab] OR "lower income countries"[tiab] OR "lower income nation"[tiab] OR

"lower income nations"[tiab] OR "lower income population"[tiab] OR "lower income populations"[tiab] OR "underserved country"[tiab] OR "underserved countries"[tiab] OR "underserved nation"[tiab] OR "underserved nations"[tiab] OR "underserved population"[tiab]

OR "underserved populations"[tiab] OR "underserved world"[tiab] OR "under served country"[tiab] OR "under served countries"[tiab] OR "under served nation"[tiab] OR "under served nations"[tiab] OR "under served population"[tiab] OR "under served populations"[tiab] OR "under served world"[tiab] OR "deprived country"[tiab] OR "deprived countries"[tiab] OR "de

prived nation"[tiab] OR "deprived nations"[tiab] OR "deprived population"[tiab] OR "deprived

populations"[tiab] OR "deprived world"[tiab] OR "poor country"[tiab] OR "poor countries"[tiab] OR "poor nation"[tiab] OR "poor nations"[tiab] OR "poor population"[tiab] OR "poor populations"[tiab] OR "poor world"[tiab] OR "poorer country"[tiab] OR "poorer countries"[tiab] OR "poorer nation"[tiab] OR "poorer nations"[tiab] OR "poorer population"[tiab] OR "poorer populations"[tiab] OR "poorer world"[tiab] OR "developing economy"[tiab] OR "developing

economies"[tiab] OR "less developed economy"[tiab] OR "less developed economies"[tiab] OR "lesser developed economy"[tiab] OR "lesser developed economies"[tiab] OR "under developed economy"[tiab] OR "under developed economies"[tiab] OR "underdeveloped economy"[tiab]

OR "underdeveloped economies"[tiab] OR "middle income economy"[tiab] OR "middle income

economies"[tiab] OR "low income economy"[tiab] OR "low income economies"[tiab] OR "lower income economy"[tiab] OR "lower income economies"[tiab] OR "low gdp"[tiab] OR "low gnp"[tiab] OR "low gross domestic"[tiab] OR "low gross national"[tiab] OR "lower gdp"[tiab] OR "lower gnp"[tiab] OR "lower gross domestic"[tiab] OR "lower gross national"[tiab] OR lmic[tiab] OR lmics[tiab] OR "third world"[tiab] OR "lami country"[tiab] OR "lami countries"[tiab] OR "transitional country"[tiab] OR "transitional countries"[tiab] OR Africa[tiab] OR Asia[tiab] OR Caribbean[tiab] OR West Indies[tiab] OR South America[tiab] OR Latin America[tiab] OR Central America[tiab] OR "Atlantic Islands"[tiab] OR "Commonwealth of Independent Stat

es"[tiab] OR "Pacific Islands"[tiab] OR "Indian Ocean Islands"[tiab] OR "Eastern Europe"[tiab] OR Afghanistan[tiab] OR Albania[tiab] OR Algeria[tiab] OR Angola[tiab] OR Antigua[tiab] OR Barbuda[tiab] OR Argentina[tiab] OR Armenia[tiab] OR Armenian[tiab] OR Aruba[tiab] OR Azerbaijan[tiab] OR Bahrain[tiab] OR Bangladesh[tiab] OR Barbados[tiab] OR Benin[tiab] OR Byelarus[tiab] OR Byelorussian[tiab] OR Belarus[tiab] OR Belorussian[tiab] OR Belorussia[tiab] OR Belize[tiab] OR Bhutan[tiab] OR Bolivia[tiab] OR Bosnia[tiab] OR Herzegovina[tiab] OR Hercegovina[tiab] OR Botswana[tiab] OR Brasil[tiab] OR Brazil[tiab] OR Bulgaria[tiab] OR Burkina Faso[tiab] OR Burkina Fasso[tiab] OR Upper Volta[tiab] OR Burundi[tiab] OR Urundi[tiab] OR Cambodia[tiab] OR Khmer Republic[tiab] OR Kampuchea[tiab] OR Cameroon

[tiab] OR Cameroons[tiab] OR Cameron[tiab] OR Camerons[tiab] OR Cape Verde[tiab] OR

Central African Republic[tiab] OR Chad[tiab] OR Chile[tiab] OR China[tiab] OR Colombia[tiab] OR Comoros[tiab] OR Comoro Islands[tiab] OR Comores[tiab] OR Mayotte[tiab] OR Congo[tiab] OR Zaire[tiab] OR Costa Rica[tiab] OR Cote d'Ivoire[tiab] OR Ivory Coast[tiab] OR Croatia[tiab] OR Cuba[tiab] OR Cyprus[tiab] OR Czechoslovakia[tiab] OR Czech Republic[tiab] OR Slovakia[tiab] OR Slovak Republic[tiab] OR Djibouti[tiab] OR French Somaliland[tiab] OR

Dominica[tiab] OR Dominican Republic[tiab] OR East Timor[tiab] OR East Timur[tiab] OR Timor Leste[tiab] OR Ecuador[tiab] OR Egypt[tiab] OR United Arab Republic[tiab] OR El Salvador[tiab] OR Eritrea[tiab] OR Estonia[tiab] OR Ethiopia[tiab] OR Fiji[tiab] OR Gabon[tiab] OR Gabonese Republic[tiab] OR Gambia[tiab] OR Gaza[tiab] OR Georgia Republic[tiab] OR Georgian Republic[tiab] OR Ghana[tiab] OR Gold Coast[tiab] OR Greece[tiab] OR Grenada[tiab] OR Guatemala[tiab] OR Guinea[tiab] OR Guam[tiab] OR Guiana[tiab] OR Guyana[tiab] OR Haiti[tiab] OR Honduras[tiab] OR Hungary[tiab] OR India[tiab] OR Maldives[tiab] OR Indonesia[tiab] OR Iran[tiab] OR Iraq[tiab] OR Isle of Man[tiab] OR Jamaica[tiab] OR Jordan[tiab] OR Kazakhstan[tiab] OR Kazakh[tiab] OR Kenya[tiab] OR Kiribati[tiab] OR Korea[tiab] OR Kosovo[tiab] OR Kyrgyzstan[tiab] OR Kirghizia[tiab] OR Kyrgyz Republic[tiab] OR Kirghiz[tiab] OR Kirgizstan[tiab] OR "Lao PDR"[tiab] OR Laos[tiab] OR Latvia[tiab] OR Lebanon[tiab] OR Lesotho[tiab] OR Basutoland[tiab] OR Liberia[tiab] OR Libya[tiab] OR

Lithuania[tiab] OR Macedonia[tiab] OR Madagascar[tiab] OR Malagasy Republic[tiab] OR

Malaysia[tiab] OR Malaya[tiab] OR Malay[tiab] OR Sabah[tiab] OR Sarawak[tiab] OR Malawi[tiab] OR Nyasaland[tiab] OR Mali[tiab] OR Malta[tiab] OR Marshall Islands[tiab] OR Mauritania[tiab] OR Mauritius[tiab] OR Agalega Islands[tiab] OR "Melanesia"[tiab] OR Mexico[tiab] OR Micronesia[tiab] OR Middle East[tiab] OR Moldova[tiab] OR Moldovia[tiab] OR Moldovian[tiab] OR Mongolia[tiab] OR Montenegro[tiab] OR Morocco[tiab] OR Ifni[tiab] OR

Mozambique[tiab] OR Myanmar[tiab] OR Myanma[tiab] OR Burma[tiab] OR Namibia[tiab] OR

Nepal[tiab] OR Netherlands Antilles[tiab] OR New Caledonia[tiab] OR Nicaragua[tiab] OR Niger[tiab] OR Nigeria[tiab] OR Northern Mariana Islands[tiab] OR Oman[tiab] OR Muscat[tiab] OR Pakistan[tiab] OR Palau[tiab] OR Palestine[tiab] OR Panama[tiab] OR Paraguay[tiab] OR Peru[tiab] OR Philippines[tiab] OR Philipines[tiab] OR Phillipines[tiab] OR Phillippines[tiab] OR

Poland[tiab] OR Portugal[tiab] OR Puerto Rico[tiab] OR Romania[tiab] OR Rumania[tiab] OR Roumania[tiab] OR Russia[tiab] OR Russian[tiab] OR Rwanda[tiab] OR Ruanda[tiab] OR Saint Kitts[tiab] OR St Kitts[tiab] OR Nevis[tiab] OR Saint Lucia[tiab] OR St Lucia[tiab] OR Saint Vincent[tiab] OR St Vincent[tiab] OR Grenadines[tiab] OR Samoa[tiab] OR Samoan Islands[tiab] OR Navigator Island[tiab] OR Navigator Islands[tiab] OR Sao Tome[tiab] OR Saudi Arabia[tiab] OR Senegal[tiab] OR Serbia[tiab] OR Montenegro[tiab] OR Seychelles[tiab] OR Sierra Leone[tiab] OR Slovenia[tiab] OR Sri Lanka[tiab] OR Ceylon[tiab] OR Solomon Islands[tiab] OR Somalia[tiab] OR Sudan[tiab] OR Suriname[tiab] OR Surinam[tiab] OR Swaziland[tiab] OR Syria[tiab] OR Syrian[tiab] OR Tajikistan[tiab] OR Tadzhikistan[tiab] OR Tadjikistan[tiab] OR

Tadzhik[tiab] OR Tanzania[tiab] OR Thailand[tiab] OR Togo[tiab] OR Togolese Republic[tiab] OR Tonga[tiab] OR Trinidad[tiab] OR Tobago[tiab] OR Tunisia[tiab] OR Turkey[tiab] OR Turkmenistan[tiab] OR Turkmen[tiab] OR Tuvalu[tiab] OR Uganda[tiab] OR Ukraine[tiab] OR Uruguay[tiab] OR USSR[tiab] OR Soviet Union[tiab] OR Union of Soviet Socialist Republics[tiab] OR Uzbekistan[tiab] OR Uzbek OR Vanuatu[tiab] OR New Hebrides[tiab] OR Venezuela[tiab] OR Vietnam[tiab] OR Viet Nam[tiab] OR West Bank[tiab] OR Yemen[tiab] OR Yugoslavia[tiab] OR Zambia[tiab] OR Zimbabwe[tiab] OR Rhodesia[tiab] OR Developing Countries[mh] OR Africa[Mesh:noexp] OR Africa, Northern[Mesh:noexp] OR Africa South of the Sahara[Mesh:noexp] OR Africa, Central[Mesh:noexp] OR Africa, Eastern[Mesh:noexp] OR Africa, Southern[Mesh:noexp] OR Africa, Western[Mesh:noexp] OR Asia[Mesh:noexp] OR Asia, Central[Mesh:noexp] OR Asia, Southeastern[Mesh:noexp] OR Asia, Western[Mesh:noexp] OR Caribbean Region[Mesh:noexp] OR West Indies[Mesh:noexp] OR South America[Mesh:noexp] OR Latin America[Mesh:noexp] OR Central America[Mesh:noexp] OR "Atlantic Islands"[Mesh:noexp] OR "Commonwealth of Independent States"[Mesh:noexp] OR "Pacific Islands"[Mesh:noexp] OR "Indian Ocean Islands"[Mesh:noexp] OR "Europe, Eastern"[Mesh:noexp] OR Afghanistan[mh] OR Albania[mh] OR Algeria[mh] OR American Samoa[mh] OR Angola[mh] OR "Antigua and Barbuda"[mh] OR Argentina[mh] OR Armenia[mh] OR Azerbaijan[mh] OR Bahrain[mh] OR "Baltic States"[mh] OR Bangladesh[mh] OR Barbados[mh] OR Benin[mh] OR "Republic of Belarus"[mh] OR Belize[mh] OR Bhutan[mh] OR Bolivia[mh] OR Bosnia-Herzegovina[mh] OR Botswana[mh] OR Brazil[mh] OR Bulgaria[mh] OR Burkina Faso[mh] OR Burundi[mh] OR Cambodia[mh] OR Cameroon[mh] OR Cape

Verde[mh] OR Central African Republic[mh] OR Chad[mh] OR Chile[mh] OR China[mh] OR Colombia[mh] OR Comoros[mh] OR Congo[mh] OR Costa Rica[mh] OR Cote d'Ivoire[mh] OR Croatia[mh] OR Cuba[mh] OR Cyprus[mh] OR Czechoslovakia[mh] OR Czech Republic[mh] OR Slovakia[mh] OR Djibouti[mh] OR "Democratic Republic of the Congo"[mh] OR "Democratic People's Republic of Korea"[mh] OR Dominica[mh] OR Dominican Republic[mh] OR East Timor[mh] OR Ecuador[mh] OR Egypt[mh] OR El Salvador[mh] OR Eritrea[mh] OR Estonia[mh] OR Ethiopia[mh] OR "Equatorial Guinea"[mh] OR Fiji[mh] OR "French Guiana"[mh] OR Gabon[mh] OR Gambia[mh] OR "Georgia (Republic)"[mh] OR Ghana[mh] OR Greece[mh] OR Grenada[mh] OR Guatemala[mh] OR Guinea[mh] OR Guinea-Bissau[mh] OR Guam[mh] OR Guyana[mh] OR Haiti[mh] OR Honduras[mh] OR Hungary[mh] OR "Independent State of Samoa"[mh] OR India[mh] OR Indonesia[mh] OR Iran[mh] OR Iraq[mh] OR Jamaica[mh] OR Jordan[mh] OR Kazakhstan[mh] OR Kenya[mh] OR Korea[mh] OR Kyrgyzstan[mh] OR Laos[mh] OR Latvia[mh] OR Lebanon[mh] OR Lesotho[mh] OR Liberia[mh] OR Libya[mh] OR Lithuania[mh] OR "Macedonia (Republic)"[mh] OR Madagascar[mh] OR Malawi[mh] OR Malaysia[mh] OR Mali[mh] OR Malta[mh] OR Mauritania[mh] OR Mauritius[mh] OR "Melanesia"[mh] OR Mexico[mh] OR Micronesia[mh] OR Middle East[Mesh:noexp] OR Moldova[mh] OR Mongolia[mh] OR Montenegro[mh] OR Morocco[mh] OR Mozambique[mh] OR Myanmar[mh] OR Namibia[mh] OR Nepal[mh] OR Netherlands Antilles[mh] OR New Caledonia[mh] OR Nicaragua[mh] OR Niger[mh] OR Nigeria[mh] OR Oman[mh] OR Pakistan[mh] OR Palau[mh] OR Panama[mh] OR Papua New Guinea[mh] OR Paraguay[mh] OR Peru[mh] OR Philippines[mh] OR Poland[mh] OR Portugal[mh] OR Puerto Rico[mh] OR "Republic of Korea"[mh] OR Romania[mh] OR Russia[mh] OR "Russia (Pre-1917)"[mh] OR Rwanda[mh] OR "Saint Kitts and Nevis"[mh] OR Saint Lucia[mh] OR "Saint Vincent and the Grenadines"[mh] OR Samoa[mh] OR Saudi Arabia[mh] OR Senegal[mh] OR Serbia[mh] OR Montenegro[mh] OR Seychelles[mh] OR Sierra Leone[mh] OR Slovenia[mh] OR Sri

Lanka[mh] OR Somalia[mh] OR South Africa[mh] OR Sudan[mh] OR Suriname[mh] OR Swaziland[mh] OR Syria[mh] OR Tajikistan[mh] OR Tanzania[mh] OR Thailand[mh] OR Togo[mh] OR Tonga[mh] OR "Trinidad and Tobago"[mh] OR Tunisia[mh] OR Turkey[mh] OR Turkmenistan[mh] OR Uganda[mh] OR Ukraine[mh] OR Uruguay[mh] OR USSR[mh] OR Uzbekistan[mh] OR Vanuatu[mh] OR Venezuela[mh] OR Vietnam[mh] OR Yemen[mh] OR Yugoslavia[mh] OR Zambia[mh] OR Zimbabwe[mh])

Other databases searched:

Embase (coverage 1966-April 23, 2015):

Same terms as keywords and used Emtree equivalents for MeSH terms.

Scopus (coverage 1996-April 23, 2015):

Searched all terms as keywords in Title-Abstract-Keyword fields.

Web of Science (coverage 1899-April 23, 2015):

Searched all terms as keywords in Topic fields.

Cochrane (coverage 1992-April 23, 2015):

Searched only triage MeSH and keywords. Limited to LMIC in title/abstract review.
