# Supplementary material for: Reliability and validity of pediatric triage tools evaluated in Low resource settings: a systematic review
Source: BMC Pediatr. 2017 Jan 26;17:37. doi: 10.1186/s12887-017-0796-x (PMC5267450; doi:10.1186/s12887-017-0796-x)
Supplement: Additional file 3: Appendix C. — Triage Tools Data Abstraction. (PDF 126 kb) [file 12887_2017_796_MOESM3_ESM.pdf]

Triage Tools Data Abstraction

Please fill out for all abstracts you intend to include. Please email Alex with any questions!

1. **Title**
   Title of Article

2. **First Author**
   Last name of first author

3. **Year**
   Mark only one oval.
   - 2000
   - 2001
   - 2002
   - 2003
   - 2004
   - 2005
   - 2006
   - 2007
   - 2008
   - 2009
   - 2010
   - 2011
   - 2012
   - 2013

4. **RID**
   Refworks ID # (0-10408)

5. **PDF Filename**
   Name of fulltext PDF file in you're DropBox folder

   ____

6. **Is the paper original research?**
   Can include RCT/Cohort study/Cross sectional study/Study on tool evaluation
   Mark only one oval.

   - ( ) Yes *Skip to question 7.*
   - ( ) No (if no explain reason under "other") *Skip to question 8.*
   - ( ) Other: ________________

Original Research

7. **What type of original research study is it?**
   Mark only one oval.

   - ( ) Case Control
   - ( ) Observational
   - ( ) Cohort
   - ( ) Randomized control trial
   - ( ) Other: ________________

Skip to question 13.

Other Paper Type

8. **What type of paper is it?**
   Mark only one oval.

   - ( ) Review (Non-Systematic) *Skip to question 9.*
   - ( ) Review (Systematic) *Skip to question 9.*
   - ( ) Letter to the Editor *Skip to question 10.*
   - ( ) Case Report *Skip to question 10.*
   - ( ) Other: ________________ *Skip to question 10.*

Skip to question 10.

Review (Systematic/Non-Systematic)

9. **Abstract**
Please copy and paste the abstract below, into the text box.

____

Skip to question 40.

Other Paper Types (Letter to editor, case report, other)

10. **Primary Country**
primary country of data collection

____

11. **Study Setting**
Type of site for investigation
Check all that apply.

- [ ] Level 3 National Hospital (Tertiary Hospital ICU, Pediatric department)
- [ ] Level 2 Provincial Hospital (Inpatient Ward, ED)
- [ ] Level 1 District Hospital (Inpatient Ward, triage area, no ED)
- [ ] Primary Clinic (Physician lead, no inpatient beds)
- [ ] Nurse lead clinic (Nurse lead, no inpatient beds)
- [ ] Health Outpost (community health workers, no inpatient beds)
- [ ] Pre hospital (EMT, pre hospital providers, etc.)
- [ ] Non Clinical
- [ ] Other: ____

12. **Triage Tool**
The triage tool evaluated, mentioned, used

____

Skip to question 40.

Location of Study

13. **Primary Country**
primary country of data collection

____

14. **Secondary Country**
If only one country, leave blank

....

15. **Study Setting**
Type of site for investigation
Check all that apply.

- [ ] Level 3 National Hospital (Tertiary Hospital ICU, Pediatric department)
- [ ] Level 2 Provincial Hospital (Inpatient Ward, ED)
- [ ] Level 1 District Hospital (Inpatient Ward, triage area, no ED)
- [ ] Primary Clinic (Physician lead, no inpatient beds)
- [ ] Nurse lead clinic (Nurse lead, no inpatient beds)
- [ ] Health Outpost (community health workers, no inpatient beds)
- [ ] Pre hospital (EMT, pre hospital providers, etc.)
- [ ] Non Clinical
- [ ] Other: ....

16. **Population density**
If multiple, please select more than one (self defined by author)
Check all that apply.

- [ ] Urban
- [ ] Rural
- [ ] Unclear

Triage Components

17. **Triage Setting**
Setting where triage takes place/purpose of triage
Check all that apply.

- [ ] Military Trauma
- [ ] Military Medical
- [ ] Civilian Trauma
- [ ] Civilian Medical
- [ ] Mass Casualty
- [ ] Natural Disaster
- [ ] Specific Complaint
- [ ] Other: ....

18. **Level of Training of Provider Performing Triage**
provider tasked with performing triage in study
Check all that apply.

- [ ] Physician
- [ ] Nurse
- [ ] EMT (formal training in emergency response)
- [ ] Medical Officer (hospital employee tasked with patient care assistance)
- [ ] Community Health Worker (volunteer or paid)
- [ ] Lay person (no health care training)

19. **Patient Age Group**
Adult, pediatric or both? (15 is used as minimum for adult, 18 as maximum for children)
Mark only one oval.

- ( ) Adults (>15 only)
- ( ) Children (<18 only)
- ( ) Both

20. **Triage Tool 1**
Enter the name of first triage tool evaluated

....

21. **Tool 1 Components of Evaluation**
Types of evaluation used in triage tool 1
Check all that apply.

- [ ] Age of patient
- [ ] Presenting Complaint
- [ ] Vital Signs
- [ ] IMCI (Integrated Management of Childhood Illness)
- [ ] TEWS (Triage Early Warning Score)
- [ ] MEWS (Modified Early Warning System)
- [ ] Clinical Discriminator
- [ ] ETAT
- [ ] Other: ....

22. **Triage Tool 2**
Enter the name of second triage tool evaluated

....

23. **Tool 2 Components of Evaluation**
Types of evaluation used in triage tool 2
Check all that apply.

- [ ] Age of patient
- [ ] Presenting Complaint
- [ ] Vital Signs
- [ ] IMCI (Integrated Management of Childhood Illness)
- [ ] TEWS (Triage Early Warning Score)
- [ ] MEWS (Modified Early Warning System)
- [ ] Clinical Discriminator
- [ ] ETAT
- [ ] Other: ....

24. **Triage Tool 3**
Enter the name of third triage tool evaluated

....

25. **Tool 3 Components of Evaluation**
Types of evaluation used in triage tool 3
Check all that apply.

- [ ] Age of patient
- [ ] Presenting Complaint
- [ ] Vital Signs
- [ ] IMCI (Integrated Management of Childhood Illness)
- [ ] TEWS (Triage Early Warning Score)
- [ ] MEWS (Modified Early Warning System)
- [ ] Clinical Discriminator
- [ ] ETAT
- [ ] Other: ....

Percentage at each triage scale

For each, please list the percent of patients (1-100) who were triaged at each level, according to the gold standard. Add multiple columns if necessary (ie for 4-5 add 4's and 5's).

26. **Emergent (Level 1, Red)**
Please list the percent of patients triaged as Emergent, per gold standard.

27. **Urgent (Level 2-3, Orange-Yellow)**
Please list the percent of patients triaged as Urgent, per gold standard.

28. **Non-Emergent (Level 4-5, Green)**
Please list the percent of patients triaged as Non-Emergent, per gold standard.

29. **Uncategorized**
Please list the percent of patients triaged as Uncategorized, per gold standard.

30. **Tool Evaluation Components?**
Did the study evaluate the reliability, validity, both or neither of the triage tools?
Mark only one oval.

- Reliability (between grader or between tool comparison) *Skip to question 31.*
- Validity (triage grade compared to outcome) *Skip to question 36.*
- Both *Skip to question 31.*
- Neither (Descriptive Study) *Skip to question 40.*
- Over and Under Triage *Skip to question 36.*
- Waiting Time *Skip to question 40.*
- Other:

Reliability Metrics

31. **What was the gold standard used?**
ie Which test (or grader) was used as the current standard for comparison?

Reliability Statistics

Statistical measures of reliability between scores or graders

32. **Percent agreement?**
If measured, what was the percent agreement between the tests?

____

33. **Kappa Statistic?**
If measured, what was the kappa statistic between the tests?

____

34. **Was validity also measured?**
Did the authors compare scores to a clinical outcome (validity)?
Mark only one oval.

- ( ) Yes
- ( ) No

35. **Any additional information from results section**

____
____
____
____
____

Validity Metrics

36. **Outcome measure?**
What outcome measure was used?
Check all that apply.

- [ ] Mortality
- [ ] General Hospitalization
- [ ] ICU Admission
- [ ] High Care
- [ ] Other: ________________________

37. **What timepoint was given for followup?**
What was the maximum time that the authors evaluated their outcome?
Mark only one oval.

- Same admission
- 1-3 days
- 3-7 days
- 1-4 weeks
- >4 weeks

38. **Outcome odds result**
Please write a sentence describing the outcome odds result obtained (ie Emergent triage grade under triage tool A had 2.5x higher odds of outcome A etc.)

39. **Any additional information from results section**

Comments

40. **Comments**
Include comments on study quality.

Powered by
Google Forms
